# “Reality shock”-a qualitative exploration of helicopter emergency medical services nurses’ challenges and experiences in China

**DOI:** 10.3389/fpubh.2025.1745515

**Published:** 2026-01-13

**Authors:** Ge Zang, Bo Li, Li Zhang, Naifu Tang, Qianqian Zhang, Zhenzhen Zhang

**Affiliations:** 1 The First Affiliated Hospital of Zhengzhou University, Zhengzhou, China; 2 Institute for Hospital Management of Henan Province, Zhengzhou, China

**Keywords:** flight nurse, helicopter emergency medical service, lived experience, professional identity, qualitative research

## Abstract

**Aim:**

To explore the lived experiences of nurses involved in Helicopter emergency medical services (HEMS), identifying the core challenges they encountered and their support needs.

**Methods:**

A qualitative research design was employed. Data were collected through semi-structured, in-depth interviews with 14 nurses who had participated in at least one HEMS mission. Colaizzi’s phenomenological method was used for data analysis to extract key themes from the participants’ narratives.

**Results:**

Four major themes emerged from the analyses: (1) *Adaptational Challenges in the Aeromedical Environment*, including operational constraints due to limited cabin space and the need for both physiological and psychological adaptation to high-altitude conditions; (2) *Multidimensional Safety Risk Management*, emphasizing the implementation of dual safety protocols for crew and patients, as well as comprehensive emergency preparedness; (3) *Professional Competency Development and Process Optimization*, identifying barriers in translating theoretical knowledge into practice, and highlighting the importance of standardized procedures and effective interdisciplinary collaboration; and (4) *Professional Identity and the Emotional Support Network*, underscoring the value of professional recognition, experience sharing, and emotional connectedness among team members.

**Conclusion:**

This study shows that most Chinese nurses encounter “reality shock” when participating in HEMS, face certain physical and mental burdens, and lack highly standardized procedures and emergency response plans during task execution. Meanwhile, their professional identity and team collaboration capabilities gradually mature as tasks progress continuously.

## 1 Introduction

In inter-hospital transfers of critically ill patients, reducing transfer time is crucial for improving treatment success rates (1–3). Aeromedical Evacuation (AE) is a specialized form of medical transport that utilizes aircraft to provide professional medical monitoring and care for patients in high-altitude environments (4–6). As a core component of aeromedical evacuation, Helicopter Emergency Medical Services (HEMS) plays an irreplaceable role in emergency medical rescue and critical patient transport by overcoming geographical barriers and significantly shortening response times (7–10, 61). The effective operation of HEMS relies on rigorous equipment maintenance, strict safety protocols, and adequate financial support (11), and it has demonstrated value in large-scale medical resource allocation during emergencies or special circumstances (12). However, this highly efficient mode of transport also presents unique clinical challenges: environmental factors during flight, such as low atmospheric pressure and hypoxia, may trigger or exacerbate respiratory distress and hemodynamic instability in patients, thereby increasing the risk of secondary complications (13, 14). This requires nurses involved to not only possess advanced critical care and emergency skills (15) but also be capable of independently managing complex clinical changes in an extreme, resource-limited, confined environment (16).

As core members of HEMS teams, nurses assume critical responsibilities including clinical monitoring, emergency response, and team collaboration (17). However, existing research has largely focused on aspects such as transfer technologies, patient outcomes, or team training (18–20), largely overlooking the lived experiences of the nurses who carry out these demanding tasks. Research indicates that air medical flight may affect nurses’ job performance (21) and that long-term involvement in aeromedical transport may exert certain impacts on nurses’ physical and mental well-being (22, 23).

In China, aeromedical transport is gradually being adopted and promoted by healthcare institutions (20). Chinese nurses typically lack prior flight experience before engaging in HEMS, rendering their physiological and psychological adaptation challenges particularly unique. Currently, no domestic studies have thoroughly investigated the psychological adaptation mechanisms or the effectiveness of organizational support among these nurses within this specialized service context. Applying qualitative research methods to explore nurses’ participation in HEMS can effectively uncover their authentic experiences and the practical challenges they encounter in this demanding environment. Therefore, this study employs a qualitative approach to explore Chinese nurses’ lived experiences in HEMS, the core difficulties they encounter, and the coping strategies they develop in response.

## 2 Design

### 2.1 Aim

To explore the experiences, challenges, and coping strategies of nurses participating in HEMS in China.

### 2.2 Design

This study is a qualitative inquiry employing a descriptive phenomenological approach, with individual interviews conducted between June 2025 and August 2025. The central research question was formulated as: “What is the experience of nurses in HEMS?” The researchers developed a thematic interview guide (24) to ensure comprehensive coverage of key topics and consistency among researchers. Based on preliminary interviews with two nurses, the interview topic guide was refined and finalized, as detailed in Table 1.

**Table 1:** Interview topic guide.

Interview Stage	Topic	Example
Introduce	Research Purpose	The purpose of this study is to understand and explore the psychological perceptions and experiences of nurses during their actual involvement in HEMS.
	Objective	The study was conducted and reported based on your psychological perceptions and experiences.
	Ethical Considerations	This conversation is recorded solely for research purposes and will be kept completely anonymous and confidential. Only the research team will have access to the data. Participation is voluntary, and participants may interrupt or withdraw from the study at any time without consequence.
Open	Introductory Questions	Could you describe the circumstances of the most memorable or your most recent involvement in a HEMS mission?
Develop	Specific Experiences	What difficulties have you encountered during your involvement in aeromedical transport? What measures do you believe could help you better cope with these challenges in aeromedical transport work?
		What difficulties have you encountered during the course of this work? What measures do you believe could help you better cope with the challenges encountered in this work?
End	Final Questions	What kind of support do you think is still needed at present?
		Do you have any other feelings or suggestions regarding HEMS that you would like to share with me?
	Confirm	Thank you for your participation, your input will be highly valuable to this research.
	Considerations	If you need any further assistance or have additional requests, please let us know.

To ensure rigor and transparency in the study, and to minimize researcher bias as much as possible, the authentic experiences of the participants were consistently maintained as the central focus. Prior to data collection, researchers engaged in writing reflective journals to document and examine their preconceived assumptions, underlying beliefs, and potential biases. During interviews, open-ended questions were employed, and researchers adopted a neutral, non-judgmental stance to elicit genuine narratives from participants. In the data analysis phase, the research team held regular peer debriefing sessions to critically review and discuss emerging interpretations, thereby enhancing the credibility and trustworthiness of the findings.

### 2.3 Research subject

This study employed purposive sampling to recruit nurses who had participated in HEMS from six tertiary hospitals located in four provinces and one municipality in China. Inclusion criteria were: (1) holding a valid nursing license and being officially registered; (2) having at least 3 years of clinical experience in critical or emergency care; (3) having completed HEMS-specific training and obtained relevant qualifications; and (4) having participated in at least one HEMS mission. Exclusion criteria included: (1) taking sick or maternity leave for more than 6 months during the study period; or (2) unwillingness to participate in the study. To ensure diversity, participants were recruited from different hospitals. The sample size was determined by the principle of data saturation, continuing recruitment until no new themes emerged from the interviews. In total, 14 nurses from six different hospitals participated in the study. Participant demographic characteristics are presented in Table 2. This study has been reviewed and approved by the Hospital Medical Ethics Committee (2024-KY-0190), and all participants provided informed consent and voluntarily took part in the research.

**Table 2:** Demographic characteristics of nurses participating in HEMS.

Serial number	Age	Sex	Marital status	Number of children	Educational background	a*	b*	c*	Clinical position	Professional title
1	44	Male	Married	2	Bachelor’s Degree	6	19	1	Clinical Nurse	Nurse-in-Charge
2	32	Male	Married	1	Bachelor’s Degree	2	8	5	Clinical Nurse	Nurse-in-Charge
3	34	Female	Married	1	Bachelor’s Degree	7	9	1	Clinical Nurse	Senior Nurse-in-Charge
4	34	Male	Married	2	Bachelor’s Degree	8	10	2	Clinical Nurse	Senior Nurse-in-Charge
5	32	Male	Married	2	Bachelor’s Degree	7	9	1	Clinical Nurse	Senior Nurse-in-Charge
6	39	Male	Married	2	Bachelor’s Degree	8	17	5	Nursing Team Leader	Senior Nurse-in-Charge
7	38	Female	Married	2	Master’s Degree	7	15	5	Nursing Team Leader	Associate Chief Nurse
8	40	Male	Married	1	Bachelor’s Degree	5	15	1	Clinical Nurse	Nurse-in-Charge
9	32	Male	Married	1	Bachelor’s Degree	3	7	2	Clinical Nurse	Nurse-in-Charge
10	31	Female	Married	1	Bachelor’s Degree	5	8	2	Nursing Team Leader	Senior Nurse-in-Charge
11	29	Male	Single	0	Bachelor’s Degree	1	4	6	Clinical Nurse	Nurse-in-Charge
12	25	Male	Single	0	Bachelor’s Degree	2	3	6	Clinical Nurse	Nurse-in-Charge
13	39	Male	Married	1	Master’s Degree	6	17	1	Nursing Team Leader	Associate Chief Nurse
14	32	Male	Married	2	Bachelor’s Degree	6	9	4	Nursing Team Leader	Senior Nurse-in-Charge

a, Years Since Obtaining the Aviation Certificate; b, Years of Participating in Critical Care Transport; c, Number of Participations in HEMS.

### 2.4 Data collection

The interviews for this study were conducted by the first author of the research team. After obtaining informed consent from each participant, all interviews were digitally audio-recorded to ensure a complete and accurate record for subsequent transcription and in-depth data analysis. Semi-structured interviews were employed as the primary data collection method. This approach was guided by a pre-designed interview guide, the content of which was closely aligned with the core variables of the study, including key topics such as “nurses’ experiences in HEMS” and “the challenges faced by nurses in this context and their coping strategies.” To prevent the interview guide from limiting the breadth and depth of information gathered, space was intentionally left for flexible follow-up questions. When participants introduced information not covered in the guide but deemed valuable to the research, the researcher pursued these emergent themes with further probing. This approach ensured a balance between collecting focused, relevant data and capturing rich, individualized experiences, thereby maintaining both thematic consistency and sensitivity to unique personal insights. To comprehensively capture meaningful information and avoid data reduction based solely on verbal content, the researcher simultaneously observed and documented participants’ nonverbal cues throughout the interviews.

Interview locations were arranged flexibly according to participants’ preferences and convenience, primarily falling into two categories: (1) In-person interviews: Conducted in a private, familiar, and distraction-free environment, a dedicated meeting room at the participant’s workplace. The researcher arrived at the site 1 hour in advance to set up two digital recording devices, check signal strength, confirm ambient noise levels (ensuring background noise remained below 30 decibels), and prepare informed consent forms and the interview guide. (2) Online interviews: For participants in distant locations or with scheduling constraints, interviews were conducted via Tencent Meeting. A technical check was performed 30 min prior to each session to ensure clear video, stable audio transmission, and privacy (e.g., preventing unauthorized access).

Interview Duration: The length of each interview was dynamically adjusted based on the complexity of the topic and the participant’s willingness to elaborate, generally ranging between 30 and 60 min. This duration aimed to cover all core sections of the interview guide within a manageable timeframe, minimizing participant fatigue. Approximately 5 min before the end of each interview, the researcher conducted an confirmation, briefly summarizing key points discussed to confirm no critical information was omitted and to allow participants the opportunity to add any previously unmentioned insights.

### 2.5 Data analysis

Within 24 h after each interview, two researchers who had participated in the data collection independently transcribed the audio recordings into verbatim textual data in Word format, while also consolidating the field notes taken during the interviews. Subsequently, another researcher from the research team, who was not involved in the transcription process, cross-checked the two transcripts for consistency. In cases where discrepancies were identified, the two transcribers first discussed and attempted to reach a consensus. If disagreements persisted after discussion, a third researcher resolved the issue by repeatedly listening to the original audio recording to determine the accurate content. This rigorous verification process ensured the authenticity and accuracy of the transcribed materials prior to thematic analysis.

The analysis of interview data was conducted using Colaizzi’s seven-step method (25). The specific implementation process is shown in Table 3. If participants indicated discrepancies, the research team revisited the data from the first step and reanalyzed as necessary until the participants confirmed the accuracy of the themes, thus ensuring the trustworthiness and credibility of the findings. Throughout the analysis, Nvivo 14 software was used to assist in data management and coding. Two researchers independently analyzed and coded the interview transcripts and each developed a preliminary set of themes. In cases where discrepancies arose between the two analyses, the research team held collaborative discussion sessions to resolve differences through consensus, thereby ensuring the objectivity and reliability of the analytical process.

**Table 3:** Colaizzi’s seven-step method specific implementation process.

Step	Step title	Action description
Step 1	Familiarization with the data	The researchers repeatedly read through the interview transcripts to become thoroughly immersed in the experiences of the 14 participants regarding HEMS, including aspects such as transport procedures, physiological and psychological responses, challenges, and needs, thereby grasping the overall context and flow of the data.
Step 2	Extraction of significant statements	Key statements related to nurses’ experiences were extracted from each transcript. For example, a participant’s statement “I suffered severe motion sickness and vomited throughout the flight; the gastrointestinal reaction was intense” was retained to ensure that the data authentically reflected lived experiences.
Step 3	Initial coding	Preliminary codes were assigned to the extracted significant statements, such as “motion sickness,” “equipment concerns,” “cramped space,” and “inadequate training,” forming an initial coding framework.
Step 4	Clustering of codes	Initial codes were grouped based on similarity into broader categories. For instance, “motion sickness,” “tinnitus,” and “noise interference” were clustered under “physiological discomfort”; “equipment battery issues” and “difficulty performing procedures in tight spaces” were grouped under “transportation environment constraints,” resulting in intermediate-level codes.
Step 5	Development of detailed descriptions	Based on the clustered codes, comprehensive descriptions were constructed by integrating participants’ original statements. Representative verbatim quotes were incorporated to enhance the authenticity and credibility of the emerging themes.
Step 6	Theme formulation	Similar theme prototypes and their descriptions were compared and synthesized repeatedly. Commonalities were identified and distilled into concise, conceptually rich phrases that captured the essence of the phenomenon, thereby refining the analysis to reveal deeper and more generalized findings.
Step 7	Theme validation	The formulated themes were returned to 8 participants for member checking, and the participants confirmed that these themes accurately reflected their experiences and intentions.

## 3 Results

**Figure 1:**
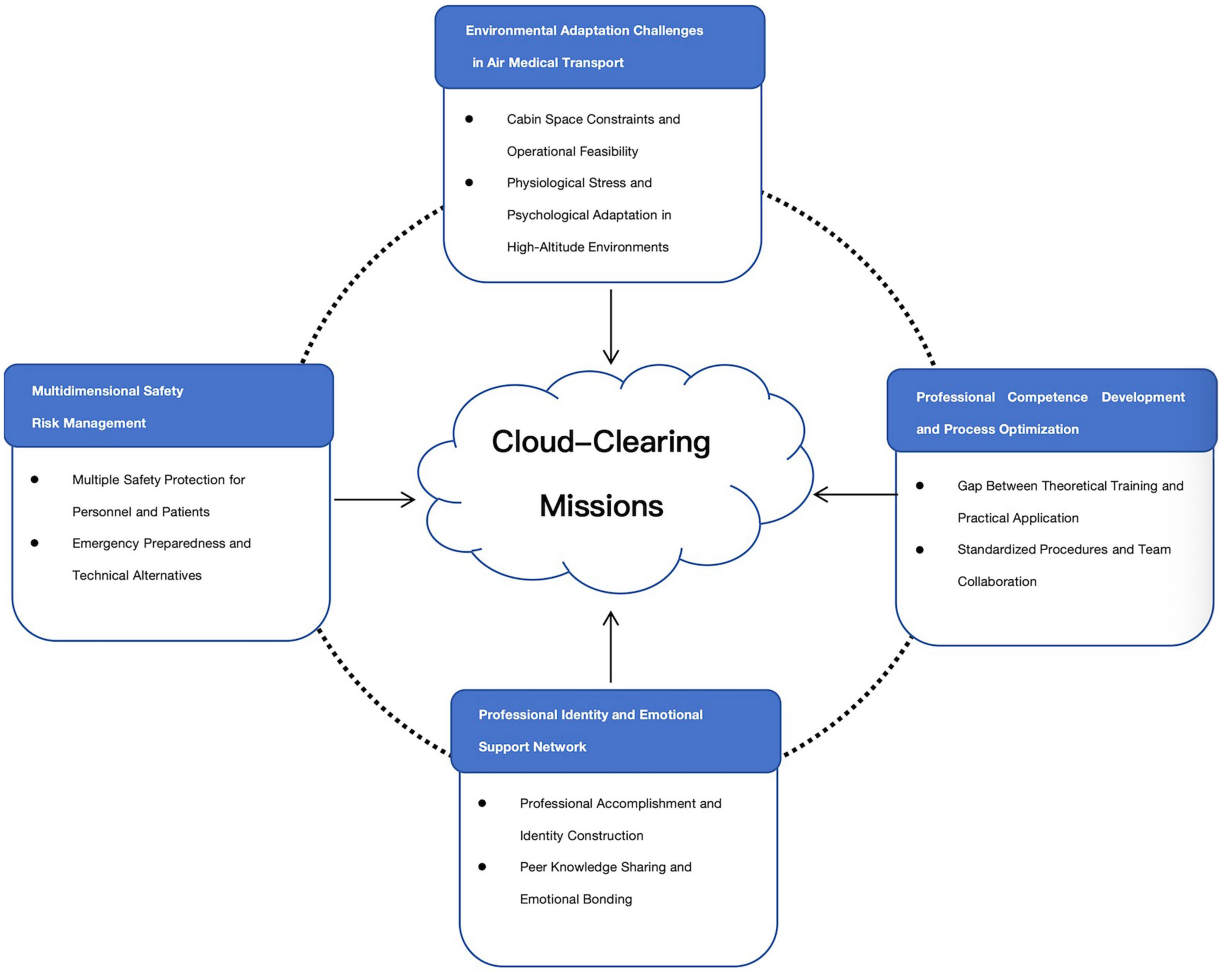
Themes and subthemes.

As illustrated in Figure 1, four core themes emerged from the analysis, each encompassing critical sub-themes that reflect the multifaceted nature of HEMS nursing. HEMS nurses must not only perform precise emergency care in the turbulent cabin of a helicopter, but also cope with high psychological pressure in extreme environments. Furthermore, they need to balance teamwork and patient care amid the fast-paced response. Their true feelings do not exist in isolation; instead, these feelings are intertwined and complement each other, collectively painting a three-dimensional portrait of this profession.

### 3.1 Theme 1: environmental adaptation challenges in air medical transport

#### 3.1.1 Subtheme 1: cabin space constraints and operational feasibility

Nurses noted that the confined space of the helicopter cabin significantly limits the feasibility and flexibility of nursing interventions, particularly imposing severe constraints on procedures requiring standing positions, such as cardiopulmonary resuscitation.

Example (P14): “*The overall layout is a bit too cramped. I feel like the space just isn't sufficient.*”

Example (P12): “*You can move around a little for instance, adjusting medications, monitoring the patient’s condition, or checking pupil response. That’s manageable. But when it comes to CPR you simply can't stand up to perform chest compressions.*”

At the same time, the dense layout of equipment in the cabin, which differs markedly from that of ground ambulances, often leads to procedural delays when nurses are unfamiliar with the location of essential items during their initial missions.

Example (P5): “*On my first transfer, I wasn't familiar with where things were kept like where are the syringes? Where are the medications? Everything felt unfamiliar.*”

This subtheme reveals the fundamental constraints that the physical environment of HEMS imposes on nursing practice. In particular, unfamiliarity with the setting during initial missions often leads to operational difficulties and delays, underscoring the critical need for training that incorporates authentic, context-specific adaptation experiences.

#### 3.1.2 Subtheme 2: physiological stress and psychological adaptation in high-altitude environments

Nurses reported that physical symptoms such as chest tightness, tinnitus, and nausea can impair the precision and stability of clinical procedures. At the same time, concerns about flight safety and fear of heights constitute significant psychological burdens.

Example (P9): “*It was extremely hot in June the aircraft was noisy and during longer flights I sometimes felt chest tightness.*”

Example (P1): “*I vomited the entire way, and I also had strong gastrointestinal reactions. I even felt like I needed to rush to the bathroom.*”

Example (P7): “*Because the aircraft is quite unstable and bumpy you yourself are not steady either so when you try to perform procedures your accuracy and overall control may be significantly affected.*”

Example (P4): “*I am somewhat afraid of heights. At the beginning especially when the helicopter started ascending I genuinely felt worried. I would have thoughts like, ‘What if we crash? How would I save myself?*”

To address these physiological and psychological challenges, nurses believe they can rely on protective equipment, adaptive behaviors, and self-directed psychological adjustment strategies.

Example (P8): “*I felt a bit of tinnitus at first but once we actually took off I adapted fairly quickly. I was still nervous but I just kept encouraging myself.*”

This theme reveals that nurses in high-altitude aeromedical environments simultaneously experience physical discomfort and psychological stress, suggesting that aeromedical systems should move beyond reliance on individual coping strategies and instead establish an integrated protective framework that combines environmental optimization, systematic training, and psychological support.

### 3.2 Theme 2: multidimensional safety risk management

#### 3.2.1 Subtheme 1: multiple safety protection for personnel and patients

In HEMS, nurses emphasized the need for rigorous assessment of the patient’s condition, the security of medical lines and equipment, and strict adherence to the use of dedicated safety harnesses and secure stretcher fixation. Additionally, they highlighted the importance of providing timely psychological support to conscious patients.

Example (P7): “*It is essential to assess whether the patient’s condition is stable, identify the auxiliary equipment and medications currently in use, and pay particular attention to the integrity of tubing, especially in patients with artificial airways.*”

Example (P11): “*Newborns on the aircraft are secured with safety belts specifically designed for infants.*”

Example (P11): “*During helicopter transport of conscious patients, the high noise levels, heat, and turbulence often cause anxiety and fear. Although hearing protection such as headphones or earplugs is provided, nurses and physicians still need to offer psychological support, for example by providing verbal reassurance or allowing the patient to hold their hand or leg.*”

Regarding the safety of healthcare personnel and bystanders, nurses emphasized that strict adherence to designated entry and exit routes must be observed, a low posture should be maintained when moving near the aircraft, and the area must be cleared during takeoff and landing to mitigate external hazards posed by the rotor blades and airflow.

Example (P6): “*The rotor blades need to be covered in training, including their height. In real helicopter transfers, we always keep our heads down to avoid being too high and getting accidentally injured. And it really matters from which angle you approach and from which angle you exit.*”

Example (P8): “*During takeoff and landing, we pay close attention to the safety of people nearby. Everyone must stay clear of the area, and any loose objects that could be blown away by the wind must be removed or secured.*”

This subtheme underscores that safety protection in helicopter aeromedical transport is a systemic endeavor encompassing personnel, equipment, procedural protocols, and psychological support. A comprehensive safety framework integrating structured physical safeguards, rigorous environmental controls, and timely psychological interventions is essential to protect both patients and healthcare providers throughout the mission.

#### 3.2.2 Subtheme 2: emergency preparedness and technical alternatives

In helicopter medical transport, nurses consider the development and implementation of systematic emergency response plans essential. However, the current coverage of these plans remains insufficient.

Example (P13): “*During the transfer, we communicate with the doctor about what emergencies might occur with this patient and how we should coordinate to manage them*.”

Example (P12): “*For neonates and pregnant women, there don’t seem to be established aeromedical emergency protocols yet. Our team currently only includes nurses trained in emergency and critical care.*”

Moreover, due to the severely limited space in the aircraft cabin, conventional emergency interventions cannot be performed and must be replaced by specialized technical equipment, for which availability must be promptly assessed.

Example (P12): “*For cardiopulmonary resuscitation, we bring a machine because manual chest compressions are not really feasible on the aircraft.*”

Example (P5): “*From a nursing perspective, we need to assess whether the onboard equipment has sufficient power, confirm that the devices are in standby mode, and verify that power sources are adequate.*”

This subtheme indicates that emergency response in helicopter medical transport relies heavily on the dual support of systematic contingency plans and specialized emergency equipment, with their synergy essential to overcoming environmental constraints and ensuring patient safety.

### 3.3 Theme 3: professional competence development and process optimization

#### 3.3.1 Subtheme 1: gap between theoretical training and practical application

Current training programs in China are characterized by a disconnect between theory and practice, with a notable lack of hands-on experience in real flight environments.

Example (P9): “*In actual training they mainly covered some conceptual content. We did go into the cabin but never actually went on a test flight.*”

Example (P13): “*The real gap I think is that we never got to practice in real operations. During training we weren’t actually given the chance to fly and experience it firsthand what problems might arise on the aircraft and how to handle them.*”

In the nurses’ view, competence development depends on the continuous accumulation of experience.

For example (P7): “*After transporting patients these few times I feel I’ve become more familiar with the cabin environment and more skilled at performing procedures onboard.*”

Example (P12): “*Honestly, most of our practical skills have been refined gradually through actual flight participation. It’s not something that gets perfected during initial training—you really learn by doing. I believe many healthcare providers can excel in aeromedical rescue, but it takes repeated exposure and practice.*”

The pathway of professional competence development moves from theory to practice, with knowledge generated through the interaction between the body and the environment (26). This subtheme reveals that healthcare personnel are prone to experiencing “reality shock” during their first operational mission due to unfamiliarity with the actual working environment.

#### 3.3.2 Subtheme 2: standardized procedures and team collaboration

Nurses believe that teams should improve work quality by developing and implementing standardized protocols. Clear and efficient team collaboration is essential to ensuring the successful execution of missions.

Example (P9): “*After every flight, we hold a debriefing session to review the entire process, summarize any issues, identify shortcomings, and determine what needs attention next time.*”

Example (P11): “*All supplies in our team are standardized and fixed. Medications can be added temporarily based on the patient’s condition. We have a designated aeromedical emergency kit with a QR code that can be scanned for pre-departure verification. In addition, we also perform manual checks by going through each item one by one before takeoff. Unlike ground transport if anything is missing or forgotten during an air mission it cannot be replaced mid-flight so thorough preparation beforehand is absolutely essential.*”

However, in the absence of standardized protocols, delays in procedures and coordination breakdowns may occur, compromising overall efficiency and patient safety.

Example (P2): “*Our team didn't have a well-established workflow or communication protocol so it caused some issues. For instance, once, due to a communication problem, the patient had to wait longer than necessary before boarding. But later our department held joint discussions and debriefings and gradually improved the situation.*”

This subtheme indicates that standardized protocols and team collaboration are fundamental to ensuring the safety and efficiency of HEMS. Standardized procedures, such as debriefing mechanisms and digital checklists, effectively reduce human-related risks.

### 3.4 Theme 4: professional identity and emotional support network

#### 3.4.1 Subtheme 1: professional accomplishment and identity construction

Nurses regard aeromedical transport as a highly technical and high-risk professional practice, and they gain recognition and a sense of pride from colleagues, family, and themselves through successfully completing missions and sharing their experiences.

Example (P4): “*Sharing my flight experiences with colleagues helps everyone gain a better understanding of what actually happens during air medical missions. When I talk about it with my family my child looks up to me with admiration and thinks Dad is really amazing. It makes me feel like I have built a strong and heroic image in their eyes. Having taken part in helicopter medical transport truly makes me feel capable and proud.*”

Example (P3): “*At first I was afraid then I became excited and finally it turned into a little bit of pride. I felt a sense of professional reward in this area and that feeling was quite proud indeed.*”

At the same time, the unique advantages of HEMS in terms of emergency response timeliness and accessibility further strengthen nurses’ sense of meaning and purpose in their work.

Example (P9): “*In our setting the cost of ambulance transport and air medical transport does not differ much but air rescue is faster. Patients are more willing to accept it mainly because it shortens transfer time which greatly enhances safety.*”

Individuals derive meaning from their experiences through narrative (27). This subtheme illustrates that professional identity is reinforced through social engagement, as nurses seek self-understanding and positioning through interactions with themselves and with society.

#### 3.4.2 Subtheme 2: peer knowledge sharing and emotional bonding

Nurses believe that sharing experiences among team members effectively reduces fear and anxiety among novice colleagues.

Example (P5): “*Every colleague who has attended the training may potentially participate in helicopter transfers. We let them know in advance what it feels like to be on the aircraft, helping to reduce fear before their first flight.*”

Example (P14): “*We also communicate with our colleagues about where to place each piece of equipment most reasonably. We discuss how to manage the situation if the patient’s condition changes. In addition, we inform them in advance that the aircraft is quite noisy, so they can be prepared ahead of time.*”

Moreover, by jointly discussing challenges encountered in practice and strategies for managing them, nurses not only refine workflows but also strengthen their collective identity and psychological resilience.

Example (P11): “*After completing flights, we exchange experiences and share what happened during the mission, including any unexpected situations and how they were handled, to avoid similar issues in the future. If a particularly noteworthy or typical case arises, we often discuss it during meetings and sometimes present it using a PPT for further sharing and learning.*”

This subtheme highlights that informal emotional exchanges are crucial for sustaining professional resilience. Participants cultivated a supportive culture through sharing experiences and empathizing with one another’s emotions. This informal support mechanism compensates for the limitations of formal training and proves particularly effective in managing psychological stress.

## 4 Discussion

This study employed semi-structured interviews to systematically explore the multidimensional factors underlying nurses’ professional adaptation and psychological experiences in HEMS. The findings indicate that the challenges and stressors encountered during aeromedical transport result from the complex interplay of individual experience, organizational support, training systems, and the external operational environment.

### 4.1 Challenges and optimization directions of special air transport environments on medical adaptability

HEMS plays an irreplaceable role in the inter-regional care of critically ill patients; however, its safety and efficiency are significantly constrained by the physical environment of the aircraft cabin. Previous studies have shown that limited space not only restricts the deployment of medical equipment and the feasibility of critical interventions such as cardiopulmonary resuscitation (28), but also that continuous vibration during flight can impair hand–eye coordination through bone conduction (29). Additionally, high-intensity noise inside the cabin not only hinders team communication but also activates the sympathetic nervous system, leading to increased heart rate and reduced attentional focus (30). The present study corroborates these findings and further reveals that these multiple stressors collectively constitute a complex physical and psychological burden on healthcare personnel. When compounded by the urgency of missions, this burden undermines both procedural precision and clinical decision-making.

To systematically address these challenges, the aviation industry has already made efforts to strengthen risk management protocols (31). Future initiatives in HEMS should further integrate engineering optimization with cognitive support strategies, such as applying human factors design principles to improve cabin layout and incorporating voice prompts or visual aids, to establish a synergistic environment–human–technology work system that maximizes the life-saving potential of aeromedical rescue. At the same time, aeromedical systems must integrate environmental optimization, systematic training, and psychological support to truly safeguard healthcare providers’ occupational safety and the quality of patient care.

### 4.2 Practical dilemmas and countermeasures in multi-dimensional management of air transport safety risks

Safety protection is a systemic endeavor encompassing physiological, psychological, physical, and environmental dimensions (32). Clinical practice must be integrated with a deep understanding of the high-altitude physiological risks to patients, as the aeromedical environment poses multidimensional threats to vulnerable populations, such as triggering complications in patients with cognitive impairment (33, 34), adversely affecting trauma patient outcomes (35), altering endotracheal tube cuff pressure (36, 37), and increasing the risk of device-related adverse events associated with advanced life support systems like ECMO (38). Moreover, in special contexts like infectious disease outbreaks, environmental safety control becomes especially crucial with appropriate airflow distribution and operational management forming the foundation for protecting both patients and healthcare providers (39, 40). Interview findings from this study show that nurses place high importance on physical securing measures, such as infant-specific safety restraints, and on avoiding external hazards through standardized protocols for entering and exiting the aircraft. At the same time, providing immediate psychological support has become a critical aspect of care in response to fear experienced by conscious patients due to environmental stressors such as noise and turbulence. Therefore, effective safety protection must combine proactive operational interventions with systematic anticipation of patients’ pathophysiological responses to the high-altitude environment, establishing an integrated safeguarding framework that encompasses physical restraint, physiological monitoring, and psychological support.

To systematically address the aforementioned risks, it is necessary to establish a comprehensive safety framework across multiple dimensions, including emergency preparedness, equipment optimization, and team collaboration. This includes establishing standardized protocols for advanced airway management (41) optimizing and adapting blood product carriage (42) and equipping aircraft with essential devices highlighted by nurses in this study such as infusion pumps and mechanical CPR machines that reduce manual workload while ensuring consistent compression quality. HEMS emergency response plans should be developed around technical standardization, personnel training, multi-system collaboration, equitable service delivery, and technological innovation, and must be dynamically updated to accommodate emerging technologies such as 5G and the demands of public health emergencies.

### 4.3 The “reality shock” poses dual challenges to both professional competence and team collaboration

Professional competence development among aeromedical nurses is highly dependent on contextualized practice. Research indicates that direct involvement in patient care is pivotal for skill acquisition (43) and that coaching-style guidance facilitates the transition from technical proficiency to holistic competence (44). This study finds that due to constraints such as high operational costs stringent safety regulations limited resource allocation and training models that prioritize theoretical instruction most Chinese nurses have no prior helicopter experience before their first patient transport mission. Consequently, they commonly encounter a “reality shock” during initial flights, a phenomenon consistent with Hakvoort’s findings (45) where previously acquired competencies fail to translate effectively into real-world practice. This suggests that the participants’ subjective sense of situational realism and emotional engagement cannot be fully replicated by enhancing equipment alone (46). Therefore this study argues that training systems should shift toward progressive experiential designs emphasizing psychological realism for example by integrating virtual reality simulations and targeted soft-skills training (47–49) to enable effective knowledge-to-competence transfer. Additionally, research shows that collective debriefing can significantly shorten reflection time (50), which offers a feasible strategy for resource-constrained aeromedical training programs.

The safety and efficiency of aeromedical operations rely on systematic workflows and collaborative mechanisms. Evidence suggests that standardized procedures such as structured handovers enhance reliability in complex scenarios (51–53). Adapting aviation-derived systemic safety frameworks (54) enables proactive risk mitigation through real-time feedback representing a dual optimization of both procedural and cognitive systems. In this study nurses’ use of standardized practices including post-flight debriefings and digital checklists not only regulated clinical actions but also created structured iterative learning environments in which individual experiences were transformed into shared team knowledge. Nurses’ situational judgment and teamwork, developed through managing dynamic risks, in turn enrich the content of operational protocols and drive their evolution toward greater flexibility and adaptability, creating a co-evolutionary cycle between competence and process.

### 4.4 The mutual empowerment of professional identity and emotional connection

In this study, professional identity stems from nurses’ lived experience and internal affirmation of the core value of saving time and sustaining life. This identity is continually reinforced through patient feedback and social sharing following successful missions, thereby influencing their sense of professional meaning (55, 56) and intention to remain in the profession (57). Nurses’ practices such as recounting their experiences to family members and expressing a desire to learn about patients’ post-transfer outcomes reflect their reliance on external validation to confirm the clinical value of their work. This identification extends beyond individual narrative construction and evolves within the team into a shared sense of mission thereby enhancing group cohesion and collective self-efficacy.

At the same time, emotional bonding and peer knowledge sharing function as essential mechanisms for managing occupational stress and maintaining psychological resilience. Peer learning (58) and reflective communication (44) not only facilitate the transfer of technical skills but also establish an emotional support network that enables individuals to reframe challenges even setbacks as opportunities for growth. Research shows that the work culture within HEMS teams emphasizes collaboration and autonomous coping (59) and that psychological support is pivotal to sustaining professional well-being (60). Therefore establishing institutionalized psychological support systems and cultivating a safe environment for emotional expression are fundamental to building a resilient professional ecosystem and ensuring the long-term stability and operational quality of aeromedical teams.

Furthermore, we recognize that the four identified themes are interrelated. Specifically, challenges related to environmental adaptation directly influence safety management practices. For example, performing clinical tasks in a confined cabin necessitates specific safety protocols to protect both healthcare personnel and patients. To overcome these environmental constraints, nurses must continuously enhance their professional competence, particularly their ability to respond effectively during emergencies. Additionally, support from colleagues, understanding from family members, and access to professional psychological counseling all contribute to nurses’ capacity to cope with these demands. Ongoing training and education further strengthen nurses’ safety awareness and technical proficiency, thereby effectively mitigating operational risks.

## 5 Conclusion

The analysis revealed four primary themes: (1) Adaptational Challenges in the Aeromedical Environment, (2) Multidimensional Safety Risk Management, (3) Professional Competency Development and Process Optimization, and (4) Professional Identity and the Emotional Support Network. This study provides an in-depth exploration of the core challenges and support needs faced by flight nurses in China, offering valuable insights for optimizing training systems, improving working conditions, and enhancing occupational health. Aeromedical nursing is a highly complex professional practice whose success depends on the synergy of technical competence, psychological adaptation, and team collaboration. Building on these findings, future efforts should focus on advancing human-factors-based cabin design, developing intelligent safety support systems, implementing integrated training models that address both physiological and psychological demands, and establishing institutionalized mental health support mechanisms to ultimately improve care quality and ensure professional sustainability.

### 5.1 Limitations and future directions

This study has several limitations. First, the sample was drawn from aeromedical operations in only certain regions of China and therefore may not fully represent the national landscape of air medical transport. Second, the qualitative interview approach, while enabling deep exploration of individual experiences, relies on subjective accounts and is thus susceptible to recall bias and social desirability effects. For example, participants may have emphasized successful cases or teamwork while downplaying errors or interpersonal conflicts. Third, the study focused exclusively on nurses’ perspectives and did not include other key stakeholders such as physicians, pilots, or dispatchers. Future research could adopt an interprofessional comparative design to uncover collaborative dynamics and operational mechanisms across roles. Finally, most interviewed nurses had limited HEMS experience; subsequent studies could observe and compare the evolving adaptation processes of novice and experienced personnel over time.

## Reporting method

The Consolidated Criteria for Reporting Qualitative Research (COREQ) reporting guideline was followed.

## Data Availability

The raw data supporting the conclusions of this article will be made available by the authors, without undue reservation.
